# *N*,*N*′-(2,2′-Dithiodi-*o*-phenyl­ene)bis­(furan-2-carboxamide)

**DOI:** 10.1107/S1600536808038828

**Published:** 2008-12-03

**Authors:** James Raftery, Hiteyeshi Lallbeeharry, Minu G. Bhowon, Sabina J. Laulloo, John A. Joule

**Affiliations:** a The School of Chemistry, The University of Manchester, Manchester M13 9PL, England; b The Chemistry Department, The University of Mauritius, Reduit, Mauritius

## Abstract

The reaction of 2,2′-dithio­bis(benzenamine) with furan-2-carbonyl chloride produced the bis-amide title compound, C_22_H_16_N_2_O_4_S_2_, which, in the crystal, formed a helix; the structure consists of two planar furanoylbenzenamines related by an improper rotation of 96.3° about the S—S bond. The *N*-furanoylbenzenamine units are planar (maximum deviations = 0.316 and 0.132 Å). Each electron-deficient acyl­furan stacks (centroid–centroid separations of the two pairs of π–π stacked aromatic rings are 3.918 and 3.953 Å) with the electron-rich benzenamine of the other *N*-furan­oyl­benzenamine unit, leading to a spiral structure. The conformation is stabilized by two bifurcated intramolecular N—H⋯(O,S) interactions.

## Related literature

For the preparation of multidentate chelating agents using 2,2′-dithio­bis(benzenamine) as starting material, see: Bhowon *et al.* (2001 ▶, 2005 ▶, 2007 ▶); Nag *et al.* (2001 ▶); Okachi *et al.* (1985 ▶); Uma & Palanaindavar (1993 ▶); Jhaumeer & Bhowon (2003 ▶).
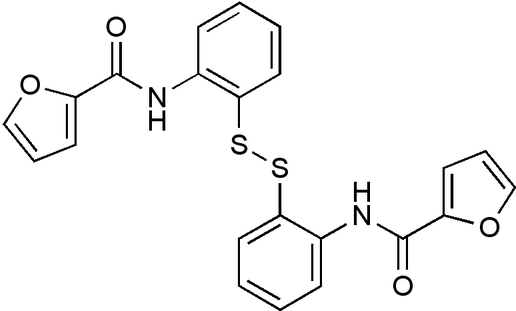


## Experimental

### 

#### Crystal data

C_22_H_16_N_2_O_4_S_2_
*M* *_r_* = 436.49
Triclinic, 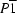

*a* = 9.6173 (11) Å
*b* = 9.9210 (11) Å
*c* = 11.9906 (14) Å
α = 109.770 (2)°
β = 103.748 (2)°
γ = 104.643 (2)°
*V* = 973.84 (19) Å^3^
*Z* = 2
Mo *K*α radiation
μ = 0.31 mm^−1^
*T* = 100 (2) K
0.45 × 0.30 × 0.20 mm

#### Data collection

Bruker SMART APEX diffractometer
Absorption correction: none
6177 measured reflections
4327 independent reflections
2911 reflections with *I* > 2σ(*I*)
*R*_int_ = 0.029

#### Refinement

*R*[*F* ^2^ > 2σ(*F* ^2^)] = 0.040
*wR*(*F* ^2^) = 0.076
*S* = 0.83
4327 reflections
271 parameters
H-atom parameters constrained
Δρ_max_ = 0.37 e Å^−3^
Δρ_min_ = −0.30 e Å^−3^

### 

Data collection: *SMART* (Bruker, 2001 ▶); cell refinement: *SAINT* (Bruker, 2002 ▶); data reduction: *SAINT*; program(s) used to solve structure: *SHELXS97* (Sheldrick, 2008 ▶); program(s) used to refine structure: *SHELXL97* (Sheldrick, 2008 ▶); molecular graphics: *SHELXTL* (Sheldrick, 2008 ▶); software used to prepare material for publication: *SHELXTL*.

## Supplementary Material

Crystal structure: contains datablocks I, global. DOI: 10.1107/S1600536808038828/ww2132sup1.cif

Structure factors: contains datablocks I. DOI: 10.1107/S1600536808038828/ww2132Isup2.hkl

Additional supplementary materials:  crystallographic information; 3D view; checkCIF report

## Figures and Tables

**Table 1:** Hydrogen-bond geometry (Å, °)

*D*—H⋯*A*	*D*—H	H⋯*A*	*D*⋯*A*	*D*—H⋯*A*
N2—H2*A*⋯S2	0.88	2.52	3.0104 (16)	116
N2—H2*A*⋯O4	0.88	2.24	2.688 (2)	111
N1—H1⋯S1	0.88	2.50	2.9805 (18)	115
N1—H1⋯O2	0.88	2.19	2.651 (2)	112

**Fig. 1.**
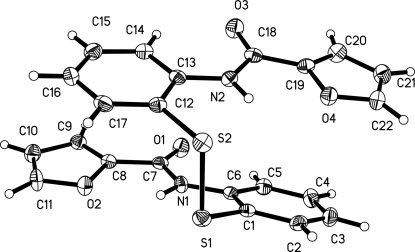
View of (I) (50% probability displacement ellipsoids).

**Fig. 2.**
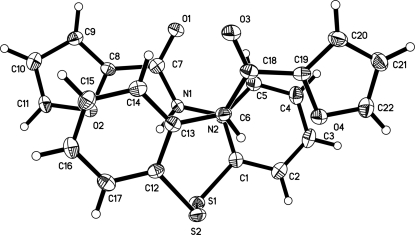
View of (I) along the S—S bond showing the sense of twist (50% probability displacement ellipsoids).

**Fig. 3.**
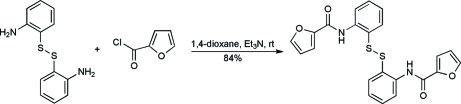
The formation of the title compound.

## supplementary crystallographic information

### Comment

We have been interested in the synthesis and properties of potential ligands which can be prepared from 2,2'-dithiobis(benzenamine). From this starting material, one can envisage the preparation of multidentate chelating agents having nitrogen, oxygen and sulfur donor atoms of relevance for the study of various mode of coordination (Okachi *et al.*, 1985; Bhowon *et al.*, 2001; Jhaumeer Laulloo & Bhowon, 2003; Bhowon *et al.*, 2007; Nag *et al.*, 2001; Uma & Palanaindavar, 1993; Bhowon *et al.*, 2005). In the present work 2,2'-dithiobis(benzenamine) *N*,*N*'-bis(furan-2-carboxamide) was synthesized (Scheme 1) with a view to examining the stereochemistry of the compound.

The structure (Figure 1) proved to be of considerable interest in adopting a helical structure, the formation of which is driven, we suggest, by the interaction of the furan and benzene units, through space. A furan ring system is normally considered electron-rich, but in this case, carrying a 2-carboxamide unit, it is electron-deficient. The benzene rings, on the other hand, each with a sulfur and a nitrogen substituent, are electron-rich, compared with an unsubstituted benzene. We suggest that interaction of these electronically opposed π-systems, through space, orients the molecule in a helix such that one acyl-furan stacks above the electron-rich benzene located at the other end of the molecule, and the other acyl-furan stacks below the other benzene ring. The second diagram, Figure 2, is a view along the S—S bond. It clearly shows the helical nature of the molecule: the molecule shown has an anticlockwise sense of twist.

The furan ring (C19—C22 and O4), the amide unit (C18, O3, N2), the benzene ring (C12—C17), and S2 are oriented in one plane, which we use as a reference plane (plane 1) (see below). The other benzene ring (C1—C6) (plane 2), the other furan ring (C8—C11 and O2) (plane 3), and the other amide unit (C7, O1, N1) (plane 4), comprise three planes at small dihedral angles one with the other: 2 *versus* 3 = 18.51°; 3 *versus* 4 = 13.75°; 2 *versus* 4 = 11.77°.

The extent to which the reference plane 1 and the three planes of the other 'half' of the molecule are not parallel is small. Quantitatively, this can be measured by the dihedral angles between plane 1 and other three planes: 1 *versus* 2 = 23.05°; 1 *versus* 3 = 8.78°; 1 *versus* 4 = 6.45°. Thus, to a close approximation, the molecule consists of two units (one planar and one close to planar) which are nearly parallel, the largest deviation from which being 23.05° (between the two benzene rings: the extent to which the planes of these can be parallel is constrained by their attachment to the disulfide unit). At the closest points, the distances between the two pairs of stacked furan and benzene aromatic rings are 2.35Å and 3.56Å.

### Experimental

A solution of 2-furoyl chloride (0.52 g, 4 mmol) in dioxane (25 ml) was stirred with triethylamine (0.25 ml) for 30 min. To the reaction mixture a solution of bis(2-aminophenyl disulfide) (0.50 g, 2 mmol) in dioxane (15 ml) was added dropwise and the mixture was stirred at room temperature for 3 h. The solution was filtered and on keeping the filtrate for 48 h a solid formed, which was filtered off, the solid washed with water and dried and gave the bis-amide (0.75 g, 84%) as yellow crystals, mp 434 K; δ_H_ (250 MHz, DMSO-d_6_) 6.52-6.54 (2H, m), 6.90-6.97 (2H, t, J 7,1 Hz), 7.17-7.18 (2H, d, J 7 Hz), 7.24-7.30 (2H, dt, J 7,1 Hz), 7.40-7.50 (4H, m), 8.45  (2H, d, J 8 Hz), 9.30 (2H, s); δ_C_ (250 MHz, DMSO-d_6_) 113.4, 115.5, 120.2, 122.8, 124.1, 132.2, 136.7, 139.6, 145.0, 147.6, 155.7; m/z (FAB) 459 (M+Na), 437 (M+H).

### Refinement

The structure was solved by direct methods. The non-hydrogen atoms were refined anisotropically. H atoms were included in calculated positions with C—H lengths of 0.95(CH), 0.99(CH_2_) & 0.98(CH_3_)Å; *U*_iso_(H) values were fixed at 1.2*U*_eq_(C) except for CH_3_ where it was 1.5*U*_eq_(C).

### Crystal data

**Table:**

C_22_H_16_N_2_O_4_S_2_	*Z* = 2
*M**_r_* = 436.49	*F*(000) = 452
Triclinic, *P*1	*D*_x_ = 1.489 Mg m^−^^3^
Hall symbol: -P 1	Melting point: 434 K
*a* = 9.6173 (11) Å	Mo *K*α radiation, λ = 0.71073 Å
*b* = 9.9210 (11) Å	Cell parameters from 1625 reflections
*c* = 11.9906 (14) Å	θ = 2.3–27.2°
α = 109.770 (2)°	µ = 0.31 mm^−^^1^
β = 103.748 (2)°	*T* = 100 K
γ = 104.643 (2)°	Plate, yellow
*V* = 973.84 (19) Å^3^	0.45 × 0.30 × 0.20 mm

### Data collection

**Table:**

Bruker SMART APEX diffractometer	2911 reflections with *I* > 2σ(*I*)
Radiation source: fine-focus sealed tube	*R*_int_ = 0.029
graphite	θ_max_ = 28.3°, θ_min_ = 1.9°
φ and ω scans	*h* = −11→12
6177 measured reflections	*k* = −12→12
4327 independent reflections	*l* = −12→15

### Refinement

**Table:**

Refinement on *F*^2^	Primary atom site location: structure-invariant direct methods
Least-squares matrix: full	Secondary atom site location: difference Fourier map
*R*[*F*^2^ > 2σ(*F*^2^)] = 0.040	Hydrogen site location: inferred from neighbouring sites
*wR*(*F*^2^) = 0.076	H-atom parameters constrained
*S* = 0.83	*w* = 1/[σ^2^(*F*_o_^2^) + (0.0233*P*)^2^] where *P* = (*F*_o_^2^ + 2*F*_c_^2^)/3
4327 reflections	(Δ/σ)_max_ < 0.001
271 parameters	Δρ_max_ = 0.37 e Å^−^^3^
0 restraints	Δρ_min_ = −0.30 e Å^−^^3^

### Special details

**Table:**

Geometry. All e.s.d.'s (except the e.s.d. in the dihedral angle between two l.s. planes) are estimated using the full covariance matrix. The cell e.s.d.'s are taken into account individually in the estimation of e.s.d.'s in distances, angles and torsion angles; correlations between e.s.d.'s in cell parameters are only used when they are defined by crystal symmetry. An approximate (isotropic) treatment of cell e.s.d.'s is used for estimating e.s.d.'s involving l.s. planes.
Refinement. Refinement of *F*^2^ against ALL reflections. The weighted *R*-factor *wR* and goodness of fit *S* are based on *F*^2^, conventional *R*-factors *R* are based on *F*, with *F* set to zero for negative *F*^2^. The threshold expression of *F*^2^ > σ(*F*^2^) is used only for calculating *R*-factors(gt) *etc*. and is not relevant to the choice of reflections for refinement. *R*-factors based on *F*^2^ are statistically about twice as large as those based on *F*, and *R*- factors based on ALL data will be even larger.

### Fractional atomic coordinates and isotropic or equivalent isotropic displacement parameters (Å^2^)

**Table:**

	*x*	*y*	*z*	*U*_iso_*/*U*_eq_	
C1	0.0310 (2)	0.5459 (2)	0.2881 (2)	0.0182 (5)	
C2	−0.0923 (2)	0.4603 (2)	0.3075 (2)	0.0222 (5)	
H2	−0.1147	0.5039	0.3816	0.027*	
C3	−0.1820 (2)	0.3115 (3)	0.2187 (2)	0.0260 (5)	
H3	−0.2684	0.2539	0.2301	0.031*	
C4	−0.1452 (2)	0.2476 (3)	0.1137 (2)	0.0254 (5)	
H4	−0.2047	0.1440	0.0547	0.031*	
C5	−0.0240 (2)	0.3304 (2)	0.0922 (2)	0.0210 (5)	
H5	−0.0006	0.2842	0.0191	0.025*	
C6	0.0639 (2)	0.4823 (2)	0.1783 (2)	0.0175 (5)	
C7	0.2528 (2)	0.5374 (2)	0.0722 (2)	0.0191 (5)	
C8	0.3733 (2)	0.6694 (2)	0.08069 (19)	0.0177 (5)	
C9	0.4744 (2)	0.6804 (2)	0.0208 (2)	0.0208 (5)	
H9	0.4866	0.5977	−0.0400	0.025*	
C10	0.5592 (2)	0.8391 (2)	0.0659 (2)	0.0269 (5)	
H10	0.6394	0.8836	0.0412	0.032*	
C11	0.5045 (2)	0.9148 (2)	0.1501 (2)	0.0276 (5)	
H11	0.5404	1.0235	0.1946	0.033*	
C12	0.4601 (2)	0.7032 (2)	0.45289 (19)	0.0175 (5)	
C13	0.4594 (2)	0.5616 (2)	0.37237 (19)	0.0175 (5)	
C14	0.5733 (2)	0.5626 (2)	0.3192 (2)	0.0205 (5)	
H14	0.5755	0.4683	0.2652	0.025*	
C15	0.6830 (2)	0.7001 (2)	0.3446 (2)	0.0247 (5)	
H15	0.7581	0.6993	0.3055	0.030*	
C16	0.6855 (2)	0.8386 (2)	0.4259 (2)	0.0249 (5)	
H16	0.7629	0.9323	0.4441	0.030*	
C17	0.5740 (2)	0.8393 (2)	0.4805 (2)	0.0221 (5)	
H17	0.5758	0.9341	0.5374	0.027*	
C18	0.3246 (2)	0.2766 (2)	0.27812 (19)	0.0193 (5)	
C19	0.1878 (2)	0.1613 (2)	0.2705 (2)	0.0193 (5)	
C20	0.1276 (2)	0.0073 (2)	0.2061 (2)	0.0259 (5)	
H20	0.1682	−0.0548	0.1537	0.031*	
C21	−0.0076 (2)	−0.0446 (2)	0.2314 (2)	0.0267 (5)	
H21	−0.0758	−0.1480	0.1991	0.032*	
C22	−0.0201 (2)	0.0812 (2)	0.3100 (2)	0.0270 (5)	
H22	−0.1007	0.0805	0.3429	0.032*	
N1	0.18360 (17)	0.57613 (18)	0.15935 (16)	0.0187 (4)	
H1	0.2194	0.6744	0.2112	0.022*	
N2	0.34384 (17)	0.42539 (18)	0.34788 (15)	0.0180 (4)	
H2A	0.2740	0.4374	0.3823	0.022*	
O1	0.21966 (16)	0.40672 (16)	−0.00702 (15)	0.0279 (4)	
O2	0.39021 (15)	0.81377 (15)	0.16313 (14)	0.0227 (3)	
O3	0.41169 (15)	0.23672 (16)	0.22600 (14)	0.0271 (4)	
O4	0.09859 (15)	0.21068 (15)	0.33633 (14)	0.0229 (4)	
S1	0.14557 (6)	0.73663 (6)	0.40340 (5)	0.02100 (14)	
S2	0.31812 (6)	0.70909 (6)	0.52536 (5)	0.02075 (14)	

### Atomic displacement parameters (Å^2^)

**Table:**

	*U*^11^	*U*^22^	*U*^33^	*U*^12^	*U*^13^	*U*^23^
C1	0.0161 (11)	0.0201 (11)	0.0206 (12)	0.0088 (9)	0.0042 (9)	0.0111 (10)
C2	0.0192 (11)	0.0301 (13)	0.0244 (13)	0.0125 (10)	0.0089 (10)	0.0160 (11)
C3	0.0201 (12)	0.0325 (14)	0.0292 (14)	0.0062 (11)	0.0092 (11)	0.0195 (11)
C4	0.0221 (12)	0.0248 (13)	0.0222 (13)	0.0013 (10)	0.0014 (10)	0.0113 (11)
C5	0.0206 (12)	0.0230 (12)	0.0165 (12)	0.0062 (10)	0.0039 (10)	0.0081 (10)
C6	0.0164 (11)	0.0209 (12)	0.0188 (12)	0.0084 (9)	0.0049 (9)	0.0124 (10)
C7	0.0189 (11)	0.0231 (12)	0.0197 (12)	0.0112 (10)	0.0064 (10)	0.0118 (10)
C8	0.0187 (11)	0.0166 (11)	0.0154 (11)	0.0071 (9)	0.0037 (9)	0.0051 (9)
C9	0.0224 (12)	0.0223 (12)	0.0209 (12)	0.0108 (10)	0.0101 (10)	0.0094 (10)
C10	0.0249 (13)	0.0296 (13)	0.0303 (14)	0.0088 (11)	0.0133 (11)	0.0157 (11)
C11	0.0293 (13)	0.0172 (12)	0.0301 (14)	−0.0004 (10)	0.0119 (11)	0.0084 (11)
C12	0.0188 (11)	0.0220 (12)	0.0135 (11)	0.0092 (9)	0.0049 (9)	0.0087 (9)
C13	0.0189 (11)	0.0192 (11)	0.0135 (11)	0.0076 (9)	0.0025 (9)	0.0078 (9)
C14	0.0213 (12)	0.0230 (12)	0.0188 (12)	0.0106 (10)	0.0072 (10)	0.0089 (10)
C15	0.0213 (12)	0.0297 (13)	0.0280 (14)	0.0111 (10)	0.0108 (11)	0.0150 (11)
C16	0.0218 (12)	0.0206 (12)	0.0317 (14)	0.0051 (10)	0.0068 (11)	0.0140 (11)
C17	0.0242 (12)	0.0183 (12)	0.0226 (13)	0.0100 (10)	0.0047 (10)	0.0080 (10)
C18	0.0217 (11)	0.0205 (12)	0.0132 (11)	0.0091 (10)	0.0025 (10)	0.0058 (9)
C19	0.0199 (11)	0.0214 (12)	0.0168 (12)	0.0104 (10)	0.0070 (10)	0.0059 (10)
C20	0.0274 (13)	0.0214 (12)	0.0229 (13)	0.0079 (10)	0.0087 (11)	0.0033 (10)
C21	0.0269 (13)	0.0189 (12)	0.0267 (14)	0.0034 (10)	0.0066 (11)	0.0065 (11)
C22	0.0232 (12)	0.0266 (13)	0.0326 (14)	0.0061 (10)	0.0116 (11)	0.0149 (11)
N1	0.0201 (9)	0.0137 (9)	0.0188 (10)	0.0031 (8)	0.0067 (8)	0.0052 (8)
N2	0.0183 (9)	0.0185 (10)	0.0170 (10)	0.0077 (8)	0.0077 (8)	0.0057 (8)
O1	0.0297 (9)	0.0186 (8)	0.0307 (10)	0.0066 (7)	0.0153 (8)	0.0035 (7)
O2	0.0251 (8)	0.0186 (8)	0.0218 (9)	0.0048 (7)	0.0122 (7)	0.0051 (7)
O3	0.0283 (9)	0.0227 (8)	0.0306 (10)	0.0107 (7)	0.0162 (8)	0.0068 (7)
O4	0.0237 (8)	0.0202 (8)	0.0244 (9)	0.0080 (7)	0.0113 (7)	0.0073 (7)
S1	0.0238 (3)	0.0201 (3)	0.0221 (3)	0.0114 (2)	0.0109 (3)	0.0080 (2)
S2	0.0248 (3)	0.0218 (3)	0.0155 (3)	0.0092 (2)	0.0085 (2)	0.0063 (2)

### Geometric parameters (Å, °)

**Table:**

C1—C2	1.392 (3)	C12—S2	1.7871 (19)
C1—C6	1.403 (3)	C13—C14	1.392 (3)
C1—S1	1.777 (2)	C13—N2	1.402 (2)
C2—C3	1.383 (3)	C14—C15	1.380 (3)
C2—H2	0.9500	C14—H14	0.9500
C3—C4	1.378 (3)	C15—C16	1.380 (3)
C3—H3	0.9500	C15—H15	0.9500
C4—C5	1.380 (3)	C16—C17	1.382 (3)
C4—H4	0.9500	C16—H16	0.9500
C5—C6	1.393 (3)	C17—H17	0.9500
C5—H5	0.9500	C18—O3	1.224 (2)
C6—N1	1.403 (2)	C18—N2	1.366 (2)
C7—O1	1.224 (2)	C18—C19	1.469 (3)
C7—N1	1.368 (2)	C19—C20	1.344 (3)
C7—C8	1.468 (3)	C19—O4	1.369 (2)
C8—C9	1.343 (3)	C20—C21	1.415 (3)
C8—O2	1.380 (2)	C20—H20	0.9500
C9—C10	1.415 (3)	C21—C22	1.340 (3)
C9—H9	0.9500	C21—H21	0.9500
C10—C11	1.341 (3)	C22—O4	1.366 (2)
C10—H10	0.9500	C22—H22	0.9500
C11—O2	1.367 (2)	N1—H1	0.8800
C11—H11	0.9500	N2—H2A	0.8800
C12—C17	1.382 (3)	S1—S2	2.0768 (8)
C12—C13	1.407 (3)		
			
C2—C1—C6	120.24 (19)	N2—C13—C12	119.02 (17)
C2—C1—S1	119.34 (17)	C15—C14—C13	120.27 (19)
C6—C1—S1	120.42 (15)	C15—C14—H14	119.9
C3—C2—C1	119.9 (2)	C13—C14—H14	119.9
C3—C2—H2	120.0	C14—C15—C16	121.12 (19)
C1—C2—H2	120.0	C14—C15—H15	119.4
C4—C3—C2	119.50 (19)	C16—C15—H15	119.4
C4—C3—H3	120.2	C15—C16—C17	119.2 (2)
C2—C3—H3	120.2	C15—C16—H16	120.4
C3—C4—C5	121.6 (2)	C17—C16—H16	120.4
C3—C4—H4	119.2	C12—C17—C16	120.5 (2)
C5—C4—H4	119.2	C12—C17—H17	119.7
C4—C5—C6	119.6 (2)	C16—C17—H17	119.7
C4—C5—H5	120.2	O3—C18—N2	125.09 (19)
C6—C5—H5	120.2	O3—C18—C19	120.47 (18)
C5—C6—N1	122.76 (19)	N2—C18—C19	114.43 (18)
C5—C6—C1	119.09 (18)	C20—C19—O4	110.13 (18)
N1—C6—C1	118.14 (18)	C20—C19—C18	131.36 (19)
O1—C7—N1	124.76 (19)	O4—C19—C18	118.50 (17)
O1—C7—C8	121.70 (19)	C19—C20—C21	106.93 (19)
N1—C7—C8	113.54 (18)	C19—C20—H20	126.5
C9—C8—O2	110.26 (18)	C21—C20—H20	126.5
C9—C8—C7	132.45 (19)	C22—C21—C20	106.23 (19)
O2—C8—C7	117.28 (17)	C22—C21—H21	126.9
C8—C9—C10	106.59 (18)	C20—C21—H21	126.9
C8—C9—H9	126.7	C21—C22—O4	110.92 (18)
C10—C9—H9	126.7	C21—C22—H22	124.5
C11—C10—C9	106.89 (18)	O4—C22—H22	124.5
C11—C10—H10	126.6	C7—N1—C6	129.57 (17)
C9—C10—H10	126.6	C7—N1—H1	115.2
C10—C11—O2	110.59 (18)	C6—N1—H1	115.2
C10—C11—H11	124.7	C18—N2—C13	129.03 (17)
O2—C11—H11	124.7	C18—N2—H2A	115.5
C17—C12—C13	120.33 (18)	C13—N2—H2A	115.5
C17—C12—S2	119.14 (15)	C11—O2—C8	105.66 (16)
C13—C12—S2	120.49 (15)	C22—O4—C19	105.80 (15)
C14—C13—N2	122.53 (18)	C1—S1—S2	103.80 (7)
C14—C13—C12	118.45 (19)	C12—S2—S1	104.78 (7)
			
C6—C1—C2—C3	0.5 (3)	C15—C16—C17—C12	−0.8 (3)
S1—C1—C2—C3	−179.76 (15)	O3—C18—C19—C20	−5.0 (4)
C1—C2—C3—C4	2.2 (3)	N2—C18—C19—C20	175.9 (2)
C2—C3—C4—C5	−2.5 (3)	O3—C18—C19—O4	176.19 (18)
C3—C4—C5—C6	0.1 (3)	N2—C18—C19—O4	−2.9 (3)
C4—C5—C6—N1	−176.52 (18)	O4—C19—C20—C21	0.4 (3)
C4—C5—C6—C1	2.6 (3)	C18—C19—C20—C21	−178.5 (2)
C2—C1—C6—C5	−2.9 (3)	C19—C20—C21—C22	−0.4 (3)
S1—C1—C6—C5	177.37 (15)	C20—C21—C22—O4	0.2 (3)
C2—C1—C6—N1	176.30 (17)	O1—C7—N1—C6	−0.5 (3)
S1—C1—C6—N1	−3.5 (2)	C8—C7—N1—C6	179.00 (18)
O1—C7—C8—C9	−6.0 (4)	C5—C6—N1—C7	−11.6 (3)
N1—C7—C8—C9	174.5 (2)	C1—C6—N1—C7	169.31 (19)
O1—C7—C8—O2	172.76 (18)	O3—C18—N2—C13	2.0 (3)
N1—C7—C8—O2	−6.8 (3)	C19—C18—N2—C13	−178.95 (18)
O2—C8—C9—C10	−0.6 (2)	C14—C13—N2—C18	3.7 (3)
C7—C8—C9—C10	178.2 (2)	C12—C13—N2—C18	−176.88 (19)
C8—C9—C10—C11	0.0 (2)	C10—C11—O2—C8	−1.0 (2)
C9—C10—C11—O2	0.6 (3)	C9—C8—O2—C11	1.0 (2)
C17—C12—C13—C14	−1.6 (3)	C7—C8—O2—C11	−178.06 (18)
S2—C12—C13—C14	−179.28 (15)	C21—C22—O4—C19	0.0 (2)
C17—C12—C13—N2	178.93 (18)	C20—C19—O4—C22	−0.3 (2)
S2—C12—C13—N2	1.2 (3)	C18—C19—O4—C22	178.75 (18)
N2—C13—C14—C15	178.88 (19)	C2—C1—S1—S2	90.48 (16)
C12—C13—C14—C15	−0.6 (3)	C6—C1—S1—S2	−89.74 (16)
C13—C14—C15—C16	2.1 (3)	C17—C12—S2—S1	88.52 (16)
C14—C15—C16—C17	−1.4 (3)	C13—C12—S2—S1	−93.76 (16)
C13—C12—C17—C16	2.3 (3)	C1—S1—S2—C12	84.72 (10)
S2—C12—C17—C16	−179.97 (16)		

### Hydrogen-bond geometry (Å, °)

**Table:**

*D*—H···*A*	*D*—H	H···*A*	*D*···*A*	*D*—H···*A*
N2—H2A···S2	0.88	2.52	3.0104 (16)	116
N2—H2A···O4	0.88	2.24	2.688 (2)	111
N1—H1···S1	0.88	2.50	2.9805 (18)	115
N1—H1···O2	0.88	2.19	2.651 (2)	112
